# Major Advances and Discoveries in Diabetes - 2019 in Review

**DOI:** 10.1007/s11892-019-1255-x

**Published:** 2019-11-04

**Authors:** Juleen R. Zierath

**Affiliations:** 1 Section of Integrative Physiology, Department of Molecular Medicine and Surgery, Karolinska Institutet, Biomedicum, Solnavägen 9, 171 65 Stockholm, SE Sweden; 2 Integrative Physiology, Department of Physiology and Pharmacology, Karolinska Institutet, Stockholm, Sweden; 3 The Novo Nordisk Foundation Center for Basic Metabolic Research, Faculty of Health and Medical Sciences, University of Copenhagen, Copenhagen, Denmark

**Keywords:** Diabetes, Metabolism, Obesity, Insulin resistance, Clinical physiology, Molecular biology

## Abstract

**Purpose:**

This review is based on a recent invited lecture at the American Diabetes Association’s 79th annual Scientific Sessions entitled “Major Advances and Discoveries in Diabetes - The Year in Review.”

**Recent Findings:**

Here I provide a written account of my presentation entitled “Major Discoveries in Diabetes over the Past Year.” I highlight several recent advances in basic science that are relevant for the diabetes field, with insight into how the key takeaways impact basic science. I also speculate on how these breakthroughs challenge the field to move the basic science “discovery” into the clinic, as well as offering a perspective on unanswered questions in relation to these advances.

**Summary:**

The review should in no way be taken as an endorsement of the highlighted work or a denunciation of any work not selected. Rather, it is a personal reflection that provides a glimpse into what is on the horizon in this rapidly evolving and exciting field!

## Introduction

Last February, I received an email from the American Diabetes Association’s (ADA) Scientific Sessions Planning Committee inviting me to participate as a speaker at the 79th annual meeting. I have always enjoyed attending the ADA Scientific Sessions, having done so each year since enrolling in my doctoral studies at Karolinska Institutet. In fact, my first scientific presentation in the diabetes field was communicated as a poster at the 50th annual meeting in 1990. Throughout my career, my interests have been focused on the pathophysiology of type 2 diabetes. In particular, on the role of epigenetic modifications in the development insulin resistance, as well the interaction between circadian rhythms and the metabolic response to exercise. The overarching goal of this research is to identify and validate molecules, pathways and ultimately new treatments that confer the benefits of exercise to improve insulin sensitivity, and attenuate the loss of skeletal muscle function with aging and type 2 diabetes. Needless to say, I was taken aback when I read the title of the proposed session - “Major Advances and Discoveries in Diabetes - The Year in Review.” This was a new concept that the program committee was exploring and they invited me and Dr. Daniel J. Drucker to be their test drivers. The session was divided into two parts to highlight “*Major Breakthroughs*” in the Clinic and at the Bench. Dr. Drucker was asked to speak about clinical developments, and I was tasked with covering advances in basic science. The session was designed to facilitate discussions between clinicians and basic scientists, with the hope of enlightening basic scientists on the latest clinical advancements and clinicians on where the field is headed in terms of bench research.

With this review, I will provide a written account of my presentation entitled “*Major Discoveries in Diabetes over the Past Year.*” I will highlight a few recent advances in basic science and give my perspective on unanswered questions and unmet needs in relationship to these advances from published literature over the last 12–18 months. Before commencing, I wish to share a few words about the process I took to select the different “*Major Breakthroughs.*” Each year, thousands of articles are written on different aspects of diabetes research and care. My initial reaction when I accepted this task was that I would never be able to go through all of this literature in the few months I had to prepare. Even if I had unlimited time to review all of the papers published over this period, any short-list would simply be my own view. Certainly another person facing the same challenge may come up with a completely different list. Diabetes is a huge field and I did not want the session to be limited to my views on this broad topic. I felt for this topic to be of interest to a wider audience, diverse views from people at different career stages and scientific interests needed to be taken into account. Consequently, I sought input from my research group and other colleagues in the field by asking “*What do you think is the major breakthrough in the field this year*”?

In the early spring, I met with my team to discuss several dozen papers highlighting potential “*Major Breakthroughs*” in diabetes research. We interrogated this literature by asking: 1) What was the question raised? 2) What did they find? 3) How does this impact basic science? 4) What was the key takeaway from the study? 5) What were the limitations? 6) What are the challenges to move the “discovery” into the clinic? In the end, we pruned the list to 19 papers. I will briefly review these papers by taking these questions into account. A caveat with shortlisting a few select papers is that I likely have missed one of your favorite discoveries or advances. Thus, I apologize in advance for any omissions. Importantly, the fact that I have selected one paper over another is by no means an endorsement of the highlighted work or a denunciation of any work not selected.

## Many Subgroups of Type 2 Diabetes

For decades, diabetes has been classified into two forms, based on the presence (type 1 diabetes) or the absence (type 2 diabetes) of autoantibodies against pancreatic islet β-cell antigens and age at diagnosis. Nevertheless, diabetes is a highly heterogeneous disorder and consequently current classifications are not sufficient for disease stratification and outcome prediction. Indeed, this is exemplified by the identification of a third subgroup of diabetes termed “latent autoimmune diabetes in adults” that is diagnosed by the presence of glutamic acid decarboxylase antibodies. Refining diabetes classification based on biomarkers and deep phenotyping may bring clinicians closer to personalized medicine and better assessment of the risk of complications at diagnosis.

To assess whether type 2 diabetes is a uniform disease and if the response to intervention can be predicted based biomarkers, Leif Groop and colleagues [1] performed a “data-driven” cluster analysis in patients with newly diagnosed diabetes from the Swedish All New Diabetics in Scania cohort. Clusters were based on six variables including glutamate decarboxylase antibodies, age at diagnosis, body mass index (BMI), glycated hemoglobin (HbA1c), and homeostatic model assessment 2 estimates of β-cell function and insulin resistance, and were related to prospective data from patient records on the development of complications and prescription of medication. The study was replicated in three independent cohorts. The authors identified five novel subgroups of adult-onset diabetes and their association with clinical outcomes including severe autoimmune diabetes (SAID); severe insulin-deficient diabetes (SIDD); severe insulin-resistant diabetes (SIRD); mild obesity-related diabetes (MOD); and mild age-related diabetes (MARD). The authors propose that the combined information from the six variables, which are central to the development of diabetes, offers a superior metric for sub-classification of type 2 diabetes versus the conventional assessment of glucose levels. They conclude that implementation of this new substratification may eventually help to tailor and target early treatment to patients, thereby representing a first step towards precision medicine in diabetes. In addition to the obvious clinical advances from this study directed towards improving the care of people living with diabetes, these findings also impact basic science. Each subgroup might differ from a genetic or epigenetic perspective, and thus understanding the biology associated with each subtype may uncover novel molecular mechanisms controlling glucose homeostasis and complications arising from diabetes. Nevertheless, this study has some limitations in that only a few markers were identified and this approach may not necessarily reveal all of the subgroups of type 2 diabetes. Other limitations include that these phenotypic variables may change throughout the disease course, such that they can only be applied at the time the clusters were derived here, i.e. soon after disease onset; and that one thing is to derive clusters from a large body of data and quite another to be able to place an individual categorically within a cluster, given the quantitative distribution of these variables in people. However, the clustering approach has direct clinical relevance and can be readily applied to existing cohorts, moving the field one step closer to personalized medicine. In addition, the stratification offers insight into the progression of disease over time and insight into risk for complications including chronic kidney disease and retinopathy. Clinicians may also use these markers to stratify patients to assess outcomes to different therapeutic interventions.

## Studying Obesity to Learn More about Thinness

Obesity is a chronic lifelong condition that results from the interaction between heritable factors with environmental influences. Excess fat accumulation due to an imbalance between energy intake and expenditure causes obesity. The increasing prevalence of obesity is recognized as a major risk for a variety of diseases including type 2 diabetes, cardiovascular disease, cancer, and musculoskeletal disorders. Given that obesity is a driving force behind the diabetes epidemic, better adherence to exercise and diet regimes, as well as safe, effective and durable weight loss therapies are needed. To this end, I. Sadaf Farooqi and colleagues [2] studied the melanocortin 4 receptor (MC4R), a brain-expressed G protein coupled receptor (GPCR) involved in weight regulation. The authors were interested in gaining a more refined understanding of MC4R signaling and its impact on clinical phenotypes. Such knowledge could inform the design of drugs targeting this pathway to treat common obesity and its complications. Earlier studies report a frameshift mutation in MC4R that reduces Gαs-mediated cyclic adenosine monophosphate accumulation is associated with dominantly inherited obesity in humans [3, 4]. Here, the authors characterized 61 MC4R variants identified in 0.5 million people from the UK Biobank and examined the associations of these variants with BMI and obesity-related cardiometabolic diseases. They found that gain-of-function variants in the MC4R gene were associated with lower BMI and lower odds of obesity, type 2 diabetes, and coronary artery disease. These variants exhibited a signaling bias for the recruitment of β-arrestin, rather than canonical Gαs-mediated cAMP production. One limitation of the study is that the functional characterization of this obesity protective variant was performed in cell culture models. Thus, the authors provide indirect evidence for a genetic association between the variant and the clinical features of the carriers. To move this discovery into the clinic, additional functional validation using in vivo models would be required to confirm the protective effects of this variant on energy homeostasis and the development of cardiometabolic impairments. Pharmacological studies are also warranted, with exploratory efforts directed towards the development of preferential agonists rather than broad spectrum agonists with a bias towards β-arrestin signaling for weight loss and for the treatment of obesity-associated metabolic disease. Overall, the authors provide insight into molecular mechanisms underpinning genetic associations with the development and protection against obesity and cardiometabolic diseases.

## New Insight into Insulin Signaling

The canonical signal transduction pathways controlling glucose metabolism involve insulin binding to the insulin receptor α-subunit, followed by autophosphorylation of the β-subunit, leading to activation of down-stream signaling events. Insulin-receptor substrates are regulatory docking proteins that associate with the insulin receptor and play a central role in the selection and differentiation of the insulin signal towards further metabolic or mitogenic events. Two papers that gained particular attention this year dealt with novel aspects of the insulin signaling cascade [5, 6]. The insulin receptor is a cell-surface receptor, however, John G. Flanagan and colleagues [5] asked whether there is a non-canonical pathway, whereby the insulin receptor play a role within the nucleus and exerts long-term effects of insulin including transcriptional regulation. They provide evidence that the insulin receptor translocates to the nuclei and interacts with transcriptional machinery at promoters, thereby identifying a novel non-canonical pathway regulating target genes highly enriched for insulin-related functions including lipid metabolism and protein synthesis and diseases including diabetes, neurodegeneration, and cancer. This finding may have relevance for type 2 diabetes, given that insulin receptor binding was impaired in an insulin-resistant disease model. However, the mechanism was mainly elucidated in HepG2 cells and *ob*/*ob* mice. Thus, further evidence from clinical material from people with diabetes studied under physiological conditions is required before fully understanding the impact of this pathway in the pathophysiology of insulin resistance in humans. Moreover, the importance of this pathway for gene regulation versus the canonical signaling pathway for metabolic regulation needs to be elucidated. Insulin receptor translocation to the nuclei is however a new pathway to enhance our understanding of insulin signaling and insulin resistance.

Mutations in the insulin receptor cause severe forms of insulin resistance [7]. Keeping with the theme of insulin receptor signaling, Nicolas Rohner and colleagues [6] used a rather unconventional model to study diabetes-associated pathologies, namely the cave-dwelling fish species *A. mexicanus* (cavefish). They found that these particular cavefish had higher fasting blood glucose levels and insulin resistant features that were attributed to a mutation in the insulin receptor that decreased insulin binding. The hyperglycemic insulin receptor mutant cavefish, paradoxically were otherwise healthy and had a normal life span. The cavefish may have acquired compensatory mechanisms to circumvent the typical deleterious effects associated with insulin resistance and hyperglycemia. In this case, reduced insulin signaling may be beneficial in a nutrient-limited environment. Despite the fact that this study was conducted in cavefish, there may be some translation to humans. The mutation identified in the insulin receptor of these cavefish is implicated in at least two known cases of the Rabson-Mendenhall syndrome, a form of severe insulin resistance in humans. A deeper analysis of these mutant cavefish might uncover an underlying evolutionary force responsible for the striking metabolic adaptations. Identification of the putative mechanisms allowing the mutant cavefish to thrive despite severe insulin resistance and hyperglycemia could be relevant for the treatment of hyperglycemia-related complications in people with diabetes.

## Breakthroughs in Islet Cell Biology

Autoimmune destruction of insulin-producing pancreatic β cells, resulting in persistent hyperglycemia, underlies the pathogenesis of type 1 diabetes. Preserving and restoring functional β-cell mass is therefore a fundamental objective of diabetes therapy. However, adult human β cells have limited regeneration potential, therefore the possibility of reprogramming other cell types into glucose-responsive, insulin-secreting β-like cells is being actively pursued. Pancreatic α cells represent a potential source of β-like cells due to their developmental similarities and their location in the pancreatic islet. Moreover, a marked decrease in α cells in mice does not affect normal glucose metabolism. Pedro L. Herrera and colleagues interrogated mechanisms regulating islet cell plasticity [8, 9]. They determined the cellular mechanisms regulating the expression of insulin in glucagon^+^ α cells with a focus on the “brake” signals [8]. They found that paracrine repressive signals originating from β and δ cells maintain the α-cell identity, with a constant repressive influence of somatostatin and insulin. Local signals drive the conversion of α-cells, such that inhibition of proximal β and δ cells leads to a substantial increase in insulin^+^ α-cell numbers. Finally, they report that α-cell conversion is only partially improved by dual inhibition of insulin and somatostatin signals, which suggests that α-cell conversion is synergistically influenced by multiple signals. These findings provide mechanistic insights into how the cell identity–differentiation equilibrium is established and advice the notion that differentiated cells maintain some “plasticity potential”. One key takeaway from this study is the finding that spontaneous insulin production in α cells is not simply due to uncontrolled stress-induced insulin gene dysregulation, but is dynamically regulated, representing a physiological compensatory response to cope with insulin insufficiency. A limitation of the work is the lack of long-term, detailed metabolic studies, and a focus primarily on rodent cells. To move this discovery into the clinic, additional studies using human cells, as well a thorough characterization of the signals that promote insulin production in α cells are warranted.

In a complementary study using islets from donors with and without diabetes they addressed the notion of “cell-identity switches”, a process in which terminally differentiated cells are converted into different cell types when stressed [9]. They found that α cells and polypeptide-producing γ cells, obtained from pancreatic islet cells from deceased human donors with and without diabetes, can be lineage traced and reprogrammed by the transcription factors PDX1 and MAFA to produce and secrete insulin in a glucose-responsive manner. The modified α cells triggered a weaker immune response when co-cultured with T cells from people with type 1 diabetes, and thus might be less likely to be destroyed than native β cells as they retain the α-cell identity. When transplanted into diabetic mice, the converted human α cells ameliorated diabetes and produced insulin for several months. These findings advance basic science in that they may lead to the identification of a gene signature to confer glucose-stimulated insulin secretion to non-β cells, possibly through small-molecule screening. This study provides conceptual evidence for islet plasticity of human cells and confirms that cell identity and maturity are flexible states. To move this “discovery” into the clinic, further work is required to elucidate pharmacological or gene therapy approaches to stimulate cell interconversion within the patient’s own pancreas, but without causing adverse effects on other cell types.

In addition to the cell reprogramming strategies highlighted above, other groups are working to restore insulin-producing β cells by immunomodulation approaches to suppress the autoimmune attack characteristic of type 1 diabetes. This is a clinically relevant approach since type 1 diabetes is characterized by pancreatic islet infiltration by autoreactive immune cells and a nearly complete loss of β cells. Using several genetically modified mouse models of severe diabetes, Rohit N. Kulkarni and colleagues [10] report that enhancing β-cell proliferation before an immune attack provides protection against the development of type 1 diabetes by preserving the immunological self-tolerance of islets through the induction of regulatory T cells. This study advances the notion that increased β-cell proliferation early in life, before immune cell invasion, prevents the progression of type 1 diabetes. While the translational significance of the findings in mouse models to human type 1 diabetes needs to be determined, the findings suggest that parental history of diabetes, or alterations secondary to obesity, type 2 diabetes, or the sex of the parent, may affect β-cell proliferation in offspring and the identity of β cells to influence the onset of type 1 diabetes in the offspring. Moving forward, future studies to determine the factors that impact the identity of β cells and trigger type 1 diabetes in the offspring are required.

## The Gut Microbiome and Metabolism

The gut microbiome has co-evolved with its host and influences health and disease throughout the lifespan. Interactions between the gut microbiota, diet, and the host have been associated with metabolic diseases including obesity, diabetes, and cardiovascular disease. Nevertheless this link is often correlative, with only a few studies investigating whether specific microbiota or microbiota-dependent metabolites can directly affect metabolic homeostasis. Microbiota-dependent metabolites in systemic circulation may influence peripheral organs and modify insulin sensitivity. Fredrik Bäckhed and colleagues [11] performed a metabolomics screen of plasma from patients with type 2 diabetes and severe obesity, and BMI-matched subjects without diabetes to identify circulating amino acid-derived metabolites. They found the concentration of imidazole propionate, a microbially produced amino acid-derived metabolite, was higher in portal and peripheral blood from the obese people with type 2 diabetes. They also reported that daily injection of imidazole propionate into germ-free mice impairs glucose tolerance and insulin signaling, suggesting imidazole propionate directly affects glucose metabolism. Using a variety of cell models, they also report that imidazole propionate inhibits insulin receptor substrate signaling through mTORC1. The key finding is the identification of imidazole propionate, a microbiota-dependent metabolite, which is coupled to the development of insulin resistance. Nevertheless, there are some caveats as this metabolite is unlikely to cause all forms of type 2 diabetes. The authors measured imidazole propionate levels in obese people with or without type 2 diabetes and a different profile may emerge for moderately overweight or normal weight people with insulin resistance or type 2 diabetes. Given that dietary factors influence the gut microbiome, clarification of the interplay between diets, bacteria and the gut environment would also be important to understand, as different macro or micro nutrients may influence the metabolomics profile in people with type 2 diabetes or obesity. Finally, to move this “discovery” into the clinic, further insight into the identity of the molecular mechanism and signaling pathways induced by imidazole propionate or other microbiota-dependent metabolites may reveal pharmacological targets to treat metabolic diseases.

## Endothelial-to-Adipocyte Extracellular Vesicle Cross-Talk

An area of fertile research is the identification of systemic factors that integrate the individual organ response with the entire body. Many researchers have been focused on identifying secreted proteins (“*tissue-kines”*) that are produced and released by various organs that exert autocrine, paracrine and/or endocrine effects on tissue-specific or whole body glucose or energy homeostasis. Philipp E. Scherer and colleagues [12] have discovered the existence of extracellular vesicle-mediated signal transduction between cell types within adipose tissue. This unexpected finding was made when the team generated an adipocyte-specific caveolin 1 knockout mouse. Caveolin 1 is a membrane-bound structural and signaling protein that is highly abundant in adipocytes and endothelial cells. Although they were successful in ablating the caveolin 1 gene from adipocytes, unexpectedly, the protein remained abundant in the cells. They found that endothelial cells in close proximity to the adipocytes delivered caveolin 1 protein in extracellular vesicle to the cells. They uncovered a network of extracellular vesicle-mediated exchange of cellular material exists in adipose tissue, whereby this exchange can replace protein levels of caveolin 1 in adipocytes despite gene disruption. This extracellular vesicle trafficking process is regulated by the systemic metabolic state and requires communication between endothelial cells in order to package the extracellular cargo into vesicles for release into the adipose tissue. The study uncovers new insight into the complex signaling mechanisms that exist among various cell types including adipocytes and vascular cells, which may also affect distal organs. The transfer of cargo packaged in these extracellular vesicles is not a passive process, but rather a physiologically regulated event, participating in the tissue response to changes in the systemic nutrient state induced by fasting/refeeding paradigms or obesity. The study has implications for the interpretation of tissue-specific knockout models and importance of single cell sequencing. While the clinical relevance may not be immediately obvious, the findings may advance the development of tissue-specific drug delivery systems by targeting endogenous extracellular vesicles or engineering biosynthetic vesicles to treat diabetes.

## New Insight into an Old Drug - Metformin

Metformin is one of the most widely prescribed drugs to treat type 2 diabetes [13]. The drug was first used to treat diabetes over 60 years ago and is currently recommended in many clinical guidelines on the management of type 2 diabetes as the first-line oral glucose-lowering agent. Metformin mainly works by inhibiting hepatic glucose production, and enhancing insulin sensitivity. Nevertheless, the precise mode of action for the glucose lowering effects of metformin remain somewhat elusive and several mechanisms have been evoked including inhibition of mitochondrial respiration, elevation of 5′-adenosine monophosphate (AMP) levels, and activation of AMP-kinase. Kei Sakamoto and colleagues [14] revisit this question and ask whether metformin targets the AMP-inhibited enzyme fructose-1,6-bisphosphatase-1 (FBP1) to lower hepatic glucose production. They found that metformin induces a mild energy stress in liver, thereby increasing the AMP concentration, which allosterically inhibits the AMP-inhibited enzyme FBP1, to lower hepatic glucose production. Using molecular approaches, they identified a point mutation in FBP1 that rendered it insensitive to AMP. Thereafter, they studied “knock-in” mice harboring the mutant form of FBP1 and found they are insensitive to metformin. The authors discovered that FBP1, a rate-controlling enzyme in gluconeogenesis, functions as a major contributor to the therapeutic action of metformin. These results are clinically relevant and suggest that FBP1 could be a key target for the treatment of type 2 diabetes, either directly using targeted inhibition, or indirectly as a consequence of inducing energy stress.

## Timing Matters - Aligning the Circadian Clock with Environmental Cues, with Implications for Chronomedicine to Treat Metabolic Diseases

The next set of “Major Discoveries” are focused on recent advances in circadian biology, including mechanism of action, impact on cellular and whole-body metabolism, and alignment of the circadian clock with diet and exercise paradigms. Circadian rhythms are controlled by central and peripheral molecular clocks that anticipate day/night cycles to optimize numerous physiological and behavioral responses over a ~24 h cycle. Perturbed circadian rhythms are associated with metabolic dysfunction, obesity, and type 2 diabetes [15]. Over the past years, there has been a surge in interest in understanding how an alignment between the molecular circadian clocks and hormone action or environmental cues (diet, exercise, and sleep) can affect metabolism.

### Insight from Structural Biology or Melatonin Receptors

Melatonin is a hormone that is involved in synchronizing circadian rhythms and regulating the sleep–wake cycle. One new advance in receptor biology, by Vadim Cherezov and colleagues [16] deals specifically with the structural biology of the melatonin receptors MT1 and MT2, two GPCRs involved in the regulation of circadian rhythm and sleep patterns. These receptors are candidates for drug development efforts to treat insomnia, circadian rhythm and mood disorders, cancer, and type 2 diabetes. The authors used mutated and stabilized MT2 to determine structural differences to MT1, as well as altered melatonin dissociation kinetics. The authors reveal insight into the molecular understanding of melatonin receptor subtype selectivity and ligand access modes, which may be important for the design of selective melatonin tool compounds and therapeutic agents that will help to dissect the melatonin system. Single-nucleotide polymorphisms associated with type 2 diabetes mapped onto MT2 structure clustered at residues near the ligand binding pocket, and receptor surface. This implies that single-nucleotide polymorphisms associated with altered melatonin sensing may be involved in the pathogenesis of type 2 diabetes, but this finding requires additional confirmation and physiological validation. The potential long-term outcome from this work may be the development of therapies targeted towards melatonin receptors for the treatment of type 2 diabetes and metabolic disease. Nevertheless, the realization of this goal may be decades away.

### Insulin Resets the Clock

Circadian rhythms are generated by an auto-regulatory transcriptional-translational feedback loop composed of positive regulators (CLOCK/BMAL1) and repressors (CRY1/2 and PER1/2) that control a large fraction of protein coding genes in mammalian cells. The central clock located in the suprachiasmatic nuclei of the hypothalamus is mainly entrained by light, while peripheral clocks respond to external cues including temperature, feeding/fasting cycles, exercise and hormones. Recent work by John S. O’Neill and colleagues [17] tested whether insulin and insulin-like growth factor 1 (IGF1), two hormones important for the control of growth and metabolism, provide signals to the molecular clock machinery in conjunction with meals. In a series of elegant work, they report that insulin and IGF1 reset circadian clocks by inducing the PER protein. The effect of insulin and IGF1 on PER protein synthesis is not restricted to any particular tissue, and facilitates circadian entrainment of gene expression, thereby providing a mechanism by which feeding cues synchronize biological clocks throughout the body. The insulin/IGF-1 induction of PER protein requires mTOR activation, increased phosphoinositide signaling and microRNA downregulation. Whether these findings have physiological relevance is still an unresolved question since the level of insulin (600 nM) that was used for many of the experiments is lethal in humans. Moreover, there is an interplay between insulin and a variety of hormones and peptides that contribute to food entrainment, and thus the effect of other nutritional cues has yet to be unraveled. Nevertheless, this work has clinical relevance since disturbing the temporal relationship between light cures and hormonal cues may be a key physiological driver underlying the association between disturbances in circadian rhythms (shift-work, jet lag) and ill health. Management of light exposure and meal timing may prevent the adverse physiological consequences of circadian disturbances on metabolic health.

### Timing of Dietary Cues

Over-nutrition/obesity has been associated with disturbed circadian rhythms and altered expression of genes controlling metabolism. Obesity may drive a vicious cycle to further impact global transcriptional and epigenomic circadian rhythms. Nevertheless, the underlying mechanism by which nutritional/dietary cues impact circadian rhythms are not well understood. Mitchell A. Lazar and co-workers, [18] studied high-fat diet-induced obese mice and reported that obesity remodeled circadian enhancers in liver, triggering synchronous high-amplitude circadian rhythms of opposing lipid pathways namely fatty acid synthesis and oxidation. The obesity-enhanced circadian transcription of lipid genes in liver required genes involved in lipogenesis (SREBP) and fatty acid oxidation (PPARα). Of interest, pharmacological lipid lowering drug therapy was more effective in the obese mice when treatment was timed at the peak expression of the nuclear receptor and fatty acid oxidation gene PPARα. The key takeaway from this study is that at least in mice, over-nutrition intensifies circadian rhythms of the non-core clock factors SREBP and PPARα in liver, such that pharmacological lipid lowering drugs are more effective when PPARα is highest. If similar results are observed in obese humans, this work may have clinical relevance. For example, it may be possible to take circadian rhythms into consideration when prescribing PPARα agonists as lipid lowering therapy to achieve optimal effects on fat oxidation and energy homeostasis. The application of “*chronomedicine*” by aligning circadian rhythms with medicine/drugs or diet/exercise/sleep regimes to manage diabetes may be a clinical reality in the future.

In keeping with the theme *chronomedicine*, Satchidananda Panda and his team have been studying the interaction between eating and timing [19]. Remarkably, they have reported that obese mice maintained on a feeding schedule in which food availability is restricted to a defined period of 8–12 h (so called *time-restricted feeding*), have sustained rhythmic expression of circadian clock components and many key metabolic regulators. Strikingly, time-restricted feeding ameliorates obesity-associated metabolic disorders compared to mice fed an isocaloric diet ad libitum [20]. In this latest work, they studied high-fat fed obese whole-body Cry1;Cry2 and liver-specific Bmal1 and Rev-erbα/β knockout mice subjected to time-restricted feeding or ad libitum diets and reported that feeding-fasting rhythms are sufficient to alleviate key metabolic diseases, including obesity and metabolic syndrome, independently of the circadian clock. Time-restricted feeding restored rhythms in feeding-fasting, metabolic and nutrient-sensing pathways and prevented fatty liver, dyslipidemia and glucose intolerance and activated cellular homeostasis maintenance pathways in clock mutant mice. This finding was unexpected given the importance of the core clock machinery in controlling gene expression. A key takeaway as from this study is that the circadian clock maintains metabolic homeostasis by sustaining daily rhythms in feeding-fasting and by maintaining balance between nutrient and cellular stress responses. The benefits of time-restricted feeding regimes in the long-term have not been assess, nor have studies been confirmed in older mice. Consequently, the durability of this dietary regime to sustain the improvements in metabolic homeostasis is unknown. In translating these findings into the clinic, it may be important to determine whether humans with circadian rhythm defects, (jet lag, shift-workers), who may be predisposed to aberrant eating patterns, as well as people with metabolic disease, can voluntarily impose a strict time-restricted eating regime to prevent or reverse metabolic diseases. The type of diet and calorie content are also other important considerations for maintaining metabolic health. For example, Bente Kiens and coworkers [21] reported saturated or polyunsaturated fat-rich diets induce extensive metabolic adaptations that maintain peripheral insulin sensitivity, lower circulating lipids, and decrease hepatic de novo lipogenesis and gluconeogenesis, enabling disposition of dietary fat without metabolic complications in humans and mice, irrespective of fatty acid type, provided caloric intake is balanced. This was an unexpected finding, but reinforces the importance of calorie balance rather than degree of fat in the diet. Thus, at least in healthy slightly overweight men, as long as calorie balance is maintained, fat-rich diets appear to be well-tolerated.

### Timing of Exercise

Time of day may be important to achieve the most robust effects of exercise training on metabolic homeostasis. Acute exercise increases glucose uptake, promotes mitochondrial biogenesis, and enhances insulin sensitivity [22], consequently regular physical exercise is recommended for the treatment and prevention of chronic conditions/diseases including obesity and diabetes [23]. Nevertheless, the most effective time of day to achieve beneficial effects on blood glucose control in people with type 2 diabetes remains unknown. In a recent study men with type 2 diabetes underwent a randomized crossover trial of two weeks morning versus afternoon high intensity interval exercise training [24]. Afternoon high intensity interval training (4 PM) was more efficacious than morning interval training (8 AM) at improving blood glucose control as assessed in the men with type 2 diabetes by continuous glucose monitors. Strikingly, morning exercise training had an acute, deleterious effect, increasing blood glucose levels, which may be related to an exaggerated counter-regulatory hormonal responses in men with type 2 diabetes after maximal dynamic exercise [25]. While the study supports the long-standing view that exercise is a critical adjunct in the management of type 2 diabetes, it highlights the importance of optimizing the timing of exercise sessions to improve glycemic control in people with type 2 diabetes. The effect of exercise training on blood glucose control at various times of day may also differ in response to exercise intensity or modality, as well as age, sex, and metabolic status (metabolically healthy, type 1 diabetes, type 2 diabetes, obesity, gestational diabetes) of the participant. Integrating regular exercise training into a daily routine, with workouts at a consistent time of day, may help to ensure that daily activity goals are met. Thus, for most people, the best time to exercise is when you can do it.

## Exercise-Responsive Systemic Factors that Control Metabolism

Exercise perturbs whole body homeostasis and ultimately every cell and organ in the body needs to adapt to the increased mechanical, metabolic, and thermoregulatory demands associated with the increased work load [26]. Exercise-responsive factors can be released from a variety of peripheral organs during work and recovery to communicate information about the status of the different tissues to maintain whole body glucose and energy homeostasis [27]. In searching for novel adipose tissue derived exercise-responsive secreted factors Laurie J. Goodyear and colleagues [28] asked whether transforming growth factor-β2 (TGF-β2) contributes to the exercise-induced improvements in metabolic homeostasis. TGF-β2 regulates embryonic development and is an immune suppressor. They found that endurance exercise training increased TGF-β2 mRNA in subcutaneous white adipose tissue from healthy young men and mice. They report TGF-β2 is an exercise-induced “adipokine” that increases with training and promotes glucose and fatty acid metabolism, presumably by normalizing inflammation. Treatment of mice for 12 days with recombinant TGF-β2 reversed the deleterious effects of high-fat diet on glucose and lipid metabolism. While the mechanism remains elusive, exercise-induced elevations in lactate appear to act as a trigger to modulate TGF-β2 expression. To move this discovery into the clinic, additional mechanistic studies are warranted, particularly in regard to the mode of action and whether any side-effects develop from TGF-β2 treatment on inflammation. Dosing will also be a factor to titer out- given that levels during exercise are transient, and levels during a protein-based therapy may be sustained. Nevertheless, the findings uncover novel molecular connections between adipose tissue and skeletal muscle in the context of exercise physiology.

Interleukin-6 (IL-6) is another exercise-responsive “*tissue-kine*” implicated in metabolic regulation [29, 30]. IL-6 is released from skeletal muscle during exercise and stimulates lipolysis. Two recent studies further biologically validate IL-6 at the level of adipose tissue as treatment for obesity [30] and at the level of the gut for the control of gastric emptying [29]. To determine the role of IL-6 on exercise-induced reductions in visceral adiposity in Krogh-Madsen and colleagues [30] performed a randomized placebo-controlled trial whereby abdominally obese adults were treated with tocilizumab (IL-6 receptor antibody) or placebo during a 12-week intervention with either bicycle exercise or no exercise. Strikingly, they found treatment with an IL-6 receptor agonist prevented the exercise-induced loss of visceral fat, indicating IL-6 is required for the training-induced reductions in adiposity. Conversely, the cardiorespiratory improvements following exercise training were IL-6-independent. In addition to the effects of IL-6 on weight loss, Helga Ellingsgaard and colleagues [29] report an acute increase in IL-6 improves glycemic in healthy and type 2 diabetic men by inhibiting gastric motility in a GLP-1-dependent manner. Treatment with the IL-6 receptor agonist also enhance gastric emptying after acute exercise in healthy men. Collectively, these two studies provide clues regarding possible therapeutic targets for treatment of obesity and insulin resistance, implicating exercise-induced IL-6 may be required to achieve weight loss and improve glucose homeostasis. The results also raise a potential concern for increased adiposity or gastric emptying in patients prescribed IL-6 receptor agonists for the treatment of some forms of arthritis. Patients receiving this type of therapy may have difficulty in preventing weight gain or promoting weight loss with exercise, or may have issues related to the mechanisms controlling neurohumoral signals that regulate gastric emptying.

## Concluding Remarks

This is an exciting time of great discovery and advances in the field of diabetology and endocrinology as many researchers are striving to move basic discoveries towards the clinic. The need for continued support of basic research is stronger than ever given the complexity associated with diabetes and obesity, the increasing number of individuals suffering with these diseases, and the long journey from discovery to treatment. If there are no discoveries emanating from basic biology, there will be no advances to translate into the clinic. Hopefully, some of the discoveries highlighted in this review will move into the clinic. Several of the *“breakthroughs”* discussed, particularly those related to diet and exercise, are already being practiced in the clinic and are important for the prevention of metabolic diseases and the promotion of healthy living. In closing, I have provided just a glimpse of what is on the horizon in this rapidly evolving and exciting field! The task has been daunting, but for every *breakthrough* highlighted, there are surely hundreds more to come! I hope you have enjoyed the year in review.
